# Isoflavones Effects on Vascular and Endothelial Outcomes: How Is the Gut Microbiota Involved?

**DOI:** 10.3390/ph17020236

**Published:** 2024-02-11

**Authors:** Samuele Laudani, Justyna Godos, Giovanni Luca Romano, Lucia Gozzo, Federica Martina Di Domenico, Irma Dominguez Azpíroz, Raquel Martínez Diaz, Francesca Giampieri, José L. Quiles, Maurizio Battino, Filippo Drago, Fabio Galvano, Giuseppe Grosso

**Affiliations:** 1 Department of Biomedical and Biotechnological Sciences, University of Catania, 95123 Catania, Italy; samulaud1991@gmail.com (S.L.); federica.didomenico@phd.unict.it (F.M.D.D.); f.drago@unict.it (F.D.); fgalvano@unict.it (F.G.); giuseppe.grosso@unict.it (G.G.); 2 Department of Medicine and Surgery, University of Enna “Kore”, 94100 Enna, Italy; giovanniluca.romano@unikore.it; 3 Clinical Pharmacology Unit/Regional Pharmacovigilance Centre, Azienda Ospedaliero Universitaria Policlinico “G. Rodolico-S. Marco”, 95123 Catania, Italy; luciagozzo86@icloud.com; 4 Research Group on Food, Nutritional Biochemistry and Health, Universidad Europea del Atlántico, Isabel Torres 21, 39011 Santander, Spain; irma.dominguez@uneatlantico.es (I.D.A.); raquel.martinez@uneatlantico.es (R.M.D.); f.giampieri@staff.univpm.it (F.G.); jlquiles@ugr.es (J.L.Q.); m.a.battino@staff.univpm.it (M.B.); 5 Universidade Internacional do Cuanza, Cuito EN250, Angola; 6 Universidad de La Romana, La Romana 22000, Dominican Republic; 7 Universidad Internacional Iberoamericana, Campeche 24560, Mexico; 8 Universidad Internacional Iberoamericana, Arecibo 00613, Puerto Rico; 9 Department of Clinical Sciences, Università Politecnica delle Marche, 60131 Ancona, Italy; 10 Department of Physiology, Institute of Nutrition and Food Technology “José Mataix”, Biomedical Research Center, University of Granada, 18016 Granada, Spain; 11 Research and Development Functional Food Centre (CIDAF), Health Science Technological Park, Avenida del Conocimiento 37, 18016 Granada, Spain; 12 International Joint Research Laboratory of Intelligent Agriculture and Agri-Products Processing, Jiangsu University, Zhenjiang 212013, China; 13 Center for Human Nutrition and Mediterranean Foods (NUTREA), University of Catania, 95123 Catania, Italy

**Keywords:** isoflavones, polyphenols, phytoestrogens, equol, vascular, gut microbiota

## Abstract

Isoflavones are a group of (poly)phenols, also defined as phytoestrogens, with chemical structures comparable with estrogen, that exert weak estrogenic effects. These phytochemical compounds have been targeted for their proven antioxidant and protective effects. Recognizing the increasing prevalence of cardiovascular diseases (CVD), there is a growing interest in understanding the potential cardiovascular benefits associated with these phytochemical compounds. Gut microbiota may play a key role in mediating the effects of isoflavones on vascular and endothelial functions, as it is directly implicated in isoflavones metabolism. The findings from randomized clinical trials indicate that isoflavone supplementation may exert putative effects on vascular biomarkers among healthy individuals, but not among patients affected by cardiometabolic disorders. These results might be explained by the enzymatic transformation to which isoflavones are subjected by the gut microbiota, suggesting that a diverse composition of the microbiota may determine the diverse bioavailability of these compounds. Specifically, the conversion of isoflavones in equol—a microbiota-derived metabolite—seems to differ between individuals. Further studies are needed to clarify the intricate molecular mechanisms behind these contrasting results.

## 1. Introduction

Oxidative stress and inflammation are pivotal players involved in the disruption of endothelial equilibrium, leading to its dysfunction and affecting the overall cardiometabolic system [1,2]. This imbalance is the main cause of the development of cardiovascular disease (CVD) and related vascular and metabolic disorders, such as obesity, diabetes, hypertension, dyslipidemia, and atherosclerotic vascular diseases [2]. For these reasons, a better understanding of the mechanisms and risk factors involved in these processes, as well as effective strategies for prevention and management are necessary. Genetic, environmental, and lifestyle factors, such as diet, play a critical role in the development of CVD [3,4]. Diets rich in plant-based foods have been reported to substantially reduce the risk of CVD [5,6,7]. One of the main mechanisms, among others, is hypothesized to depend on their content in (poly)phenol, natural compounds with a variety of properties related to their chemical composition, which can also exert effects on human health. A recent summary of evidence suggests that a higher intake of certain (poly)phenol classes is associated with a lower risk of hypertension [8], CVD [9], and CVD-related mortality [10].

A peculiar group of (poly)phenols are phytoestrogens—molecules with a chemical structure similar to estradiol—which can exert some effects on the human system through the interaction with estrogen receptors (ERs) [11]. Isoflavones, mainly represented by daidzein and genistein, are phytoestrogens that raised great interest for their potentially hormone-related relevant effects on human health [11]. Many studies have demonstrated that isoflavone intake was associated with reduced menopausal symptoms [12], decreased incidence of different types of cancers (breast and prostate cancers) [13], and osteoporosis [14,15]. Isoflavones per se have a low bioavailability, while they are metabolized by enteric cells and the gut microbiota to produce more available and active metabolites [16,17]. In particular, equol is a gut microbiota-derived metabolite showing a greater estrogenic effect than isoflavones [18,19]. However, while all animals are able to convert isoflavones in equol, human studies demonstrated that individuals may have substantially different responses to isoflavone intake (such as being equol-producers and equol-non-producers) depending on genetic factors (given the considerable inconsistencies observed by geographical area [20]. In this regard, investigation of the gut microbiota composition and the differences between equol-producers and equol-non-producers is important to understand other potential variables related to the ability to metabolize isoflavones. Improving our knowledge on this matter would allow the development of possible interventions to modify gut microbiota in order to increase equol production and the benefit derived. In this review, we will discuss the clinical evidence known to date on isoflavone interventions and vascular outcomes. Furthermore, we will sum up the implication of gut microbiota in isoflavone metabolism and the main molecular mechanisms of action of major gut-derived metabolites.

## 2. Gut Microbiota and Its Implication in Cardiovascular Disease

**Figure 1:**
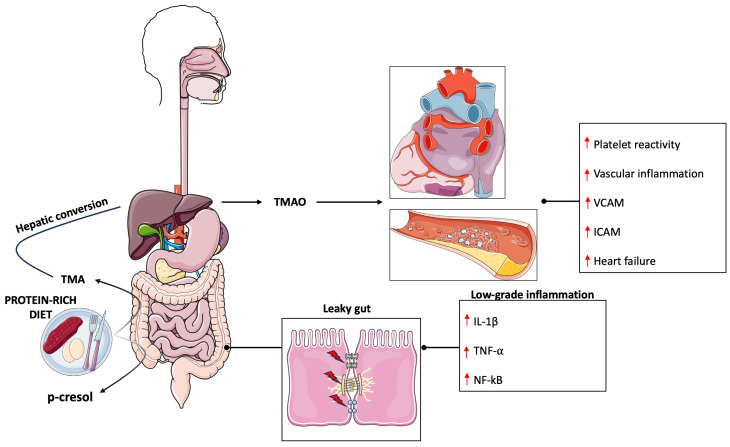
Gut-microbiota derived metabolites involved in cardiovascular disease. High protein intake leads to an increased production of TMA and p-cresol that negatively impacts gut permeability leading to systemic low-grade inflammation. IL-1beta (Interleukin-1 beta), ICAM (Intercellular Adhesion Molecule 1), NF-kB (Nuclear factor kappa B), TMA (Trimethylamine), TMAO (Trimethylamine N-Oxide), TNF-alpha (Tumor necrosis factor alpha), VCAM (Vascular cell adhesion protein). ↑ denotes increase.

The microbiota is defined as the whole microorganisms that cohabit inside/outside of a host [21]. It is composed of bacteria, archaea, viruses, and eukaryotes, although most of the studies are focused on the analysis of bacterial composition. In the last few years, researchers have moved their attention to the microbiota that inhabit the gut for its implication in health and disease [22]. The gut microbiota is composed mainly of the phyla Firmicutes and Bacteroidetes, followed by Proteobacteria and Actinobacteria [23]. Generally, a low Firmicutes/Bacteroidetes ratio is considered a health marker while on the contrary a high ratio is attributed to an unhealthy status [24,25]. More deeply, alterations in the microbiota composition have been correlated to different pathologies, such as neuropsychiatric disorders, obesity, irritable bowel disease, and CVD [21]. Oxidative stress and inflammation are risk factors involved in the pathogenesis of CVD [26,27] and gut microbiota play a key role in their regulation [28]. Indeed, a healthy microbiota is associated with reduced harmful metabolite production and tight junction integrity, which is linked to lower inflammation and oxidative stress, whereas an unhealthy microbiota is correlated with the production of harmful metabolites, such as trimethylamine (TMA) and p-cresol, increased gut permeability (leaky gut), and the onset of a low-grade inflammatory state [29,30,31] (Figure 1).

Many studies have investigated the role of the microbiota in CVD onset and pathological raise in blood pressure (BP). When comparing the microbiota composition of healthy with hypertensive and pre-hypertensive participants it was observed a significant increase in the abundance of pro-inflammatory taxa such as *Prevotella* and *Klebsiella* genera and a significant reduction of *Faecalibacterium*, *Oscillibacter*, *Roseburia*, *Bifidobacterium*, *Coprococcus*, and *Butyrivibrio.* These alterations were also associated with changes in circulating metabolites, such as increased levels of stearic acid in hypertensive patients, and possibly related to the onset of a low-grade inflammatory state [32]. Similar results have demonstrated decreased levels of *Faecalibacterium prausnitzii* and *Lachnospiraceae* family and increased levels of *Ruminococcus*, *Prevotella*, *Hungatella*, and *Succinclasticum* genera [33]. Furthermore, recent studies described a case of bacteria translocation from the gut to the heart [34] and the detection of gut microbial DNA in the plaques [35]. Gut dysbiosis is strictly correlated to an alteration in gut permeability that leads to the translocation of bacteria products into host circulation resulting in a proinflammatory state [30]. Indeed, when the gut barrier is impaired, lipopolysaccharides (LPS) can cross the intestinal lumen, reaching the circulation and binding the Toll-like receptor (TLR) on the surface of immune cells [36] triggering an inflammatory process that leads to the production of proinflammatory cytokines [37]. In a recent study, it was observed that increased LPS concentrations were predictive of major adverse cardiac events in subjects with atrial fibrillation [38]. However, it should be noted that other factors contribute to the development of CVD. In this regard, it is also important to consider that change in the microbiota composition led to an alteration in microbial-derived metabolites which could affect the physiological function of different organs. One of the most famous and discussed metabolites produced by gut bacteria in literature is TMA [39]. TMA is produced starting from different precursors, such as choline [40], phosphatidylcholine [41] and carnosine [42]. TMA reaches systemic circulation and is further metabolized in the liver by the enzyme flavin monooxygenases to produce trimethylamine N-oxide (TMAO) [43]. Circulating levels of TMAO have been associated with CVD outcome [44,45] and it was observed to promote platelet reactivity [46,47], vascular inflammation [48,49], and heart failure [50,51]. However, TMAO is not the only metabolite associated with CVD. Indeed, another candidate showed a strong association with a major adverse cardiac event identified as phenylacetylglutamine (PAG), a metabolite produced during gut phenylalanine metabolism [52]. A study conducted on 4000 participants showed an increase in the incidence of major adverse cardiac events like heart attack, stroke, and death associated with PAG [52]. PAG was shown to interact with adrenergic receptors (ARs) which are involved in heart disease and platelet functions [53,54,55]; and its detrimental effects could be attenuated by β-blocker carvedilol [52]. However, more studies are necessary to further characterize the function of PAG in host physiology.

## 3. Isoflavones Metabolism and Impact on Gut Microbiota Composition

The phytoestrogen isoflavones, of which genistein and daidzein are the main studied, are flavonoid compounds that, due to their molecular structure and size, resemble vertebrate steroid estrogen and interact with their receptors [15]. The orally ingested phytoestrogens are mainly in the form of glycosides (genistin and daidzin) that are rapidly cleaved during the passage in the intestinal lumen by the glycosidase enzyme produced by the intestinal epithelium [16] or by the bacteria in the small intestine [17] to release the aglycone. The latter is rapidly absorbed and modified in the liver to be released in the colon and further metabolized by colon bacteria [56]. Daidzein and genistein, the aglycone of daidzin and genistin, are converted by gut bacteria to produce equol or 5-OH-equol, respectively [18,19]. However, while all animals tested in in vivo experiments demonstrated to produce equol, in humans only 25–50% of subjects (named equol-producers) possess the capability to produce it while the great part of humans does not synthesize equol but convert daidzein and genistein into O-desmethylangolensin (O-DMA) and 6′-OH-O-DMA, respectively [19,20]. The inter-individual difference in equol production could be attributed to the microbiota composition. Many studies showed that bacteria with the capacity to convert daidzein into equol are largely of the *Coriobacteriaceae* family. Among these, different bacteria included in the *Eggerthella* genus [57], such as *Eggerthella julong* 732 strain [58], and the species *Adlercreutzia equolifaciens* have been observed to produce equol [59]. Other strains that have been reported to be equol-producers without being included in the *Coriobacteriaceae* family are *Lactococcus garvieae 20–92* [60] and *Pediococcus pentosaceus* [61]. Genomic analysis identified three genes in the daidzein-equol conversion which are daidzein reductase (DZNR), dihydrodaidzein reductase (DHDR) and tetrahydrodaidzein reductase (THDR) [20]. Analysis of the GC content in bacteria outside the *Coriobacteriaceae* family revealed higher GC content in these genes compared to the overall genomic GC content, suggesting that these genes were acquired through horizontal transfer [62,63]. Different studies have observed that isoflavone and isoflavone-derived metabolite supplementation can influence microbiota composition. Dietary supplementation with soy bars containing isoflavones led to an increase in *Bifidobacterium* genus with a greater increase of *Bifidobacterium* and *Eubacterium* observed in equol-producers versus non-equol-producers [64]. In another study, equol supplementation was positively correlated with an increase in *Bifidobacterium* and negatively correlated with *Clostridium cluster IV* [65]. Similar results have been observed in an in vivo study, which demonstrated that isoflavone supplementation was associated with a significant change in the microbiota composition with an increase in SCFA production and in *Bifidobacterium* spp. [66]. Similarly, in another in vivo study isoflavones administration was associated with the augmented composition of bacteria belonging to the genus *Bifidobacterium*, *Akkermansia*, *Bacteroides* and *Firmicutes* compared to the control [67]. Another study aimed to evaluate the effects of genistein supplementation on glucose metabolism and adipose tissue browning, investigated the genistein-mediated microbiota changes showing a significant increase in *Ruminiclostridium_5*, *Ruminiclostridium_9*, and *Blautia* which were significantly correlated with browning markers and glucose tolerance [68].

## 4. Clinical Evidence on the Effect of Isoflavones on Vascular Outcomes

Isoflavones, present mainly in soy and soy-based foods, represent a subclass of flavonoids that comprises a group of molecules with pleiotropic effects, including weak estrogenic activity. Various studies have demonstrated the beneficial effects of phytoestrogens on vascular and endothelial parameters (Table 1).

Several RCTs explored the effect of soy and soy-derived products, including soy legumes, soy flour, and soy protein, in both healthy and disease-affected individuals, reporting contradictory results. In a double-blind, RCT 179 healthy participants (96 men and 83 postmenopausal women) with a mean age of 62 years were supplemented with 56 g/day of powdered soy protein (providing 118 mg of total isoflavones, 75.6 mg genistein, 36.96 mg daidzein, 5.04 mg glycitein) or placebo: results showed that soy supplementation reduced mean BP (from 93 ± 1 to 87 ± 1 mmHg; *p* < 0.01), SBP (from 130 ± 2 to 123 ± 2 mmHg; *p* < 0.05) and DBP (from 76 ± 1 to 72 ± 1 mmHg; *p* < 0.01) compared to the placebo group; furthermore, it was observed a significant improvement of the femoro-dorsal PWV (from 11.1 ± 0.2 to 10.3 ± 0.2; *p* = 0.02) and a significant reduction in brachial artery FMD only in men (*p* < 0.05) compared to the placebo group [69]. In a crossover RCT, 23 healthy volunteers aged between 60 and 70 years were recruited to analyze the effects of 64 g/day of soy nuts containing 174 mg of isoflavones: after the intervention, a significant increase in FMD (*p* = 0.040) was reported in the soy nuts group compared to the control group [70]. Similar findings were reported in studies on individuals at CVD risk. A double-blind, parallel-group dietary intervention RCT evaluated the effects of 20 g/day of soy powder supplementation (providing 80 mg of isoflavones) on BP in 50 men with relatively higher BP and/or total cholesterol levels with a mean age of 52 years. Results showed that soy supplementation was able to significantly reduce SBP (from 142.0 ± 3.0 to 131.2 ± 3.1 mmHg, *p* = 0.001) and DBP (from 87.1 ± 1.8 to 82.0 ± 1.8 mmHg, *p* = 0.002) compared to baseline values, while no differences were observed for the placebo group [71]. In a double-blind, RCT it was investigated the effects of 40 g/day of soybean consumption (providing 79.4 mg total isoflavone, 44.9 mg genistein, 26.5 mg daidzein, 4.9 mg of glycitein) on BP in 302 participants (mean age of 49 years) with untreated BP for 12 weeks: results showed a significant decrease in SBP (−7.88, 95% CI: −4.66 to −11.1) and DBP (−5.27, 95% CI: −3.05 to −7.49) [72]. However, other studies conducted on healthy and unhealthy individuals using soy-derived products reached null results. A double-blind RCT, which investigated the effects of isoflavone intake among 89 hypercholesterolaemic participants with a mean age of 60 years who were randomly divided to receive 30 g/daily of soy protein (providing 100 mg of isoflavones) or 30 g/day of placebo (casein) for 24 weeks, reported no differences in FMD after treatment [73]. Furthermore, a double-blind, RCT conducted on 180 postmenopausal women (mean age 59 years) evaluated the effects of 100 mg/day of isoflavone supplementation (providing 35 mg daidzin, 59 mg genistin, 4 mg glycitin) combining soy or milk protein, or placebo on BP showed that after 6 months of treatment no differences between groups were observed [74]. In another double-blind RCT, 61 postmenopausal women (mean age 50 years) were supplemented with 33 g/day of soy-derived products, providing 54 mg of isoflavones, or placebo for 8 weeks leading to no differences were observed for SBP and DBP [75]. Similar results have been obtained from a parallel-group, double-blind, RCT conducted on 253 postmenopausal women (mean age 56 years) and supplemented with 40 g/day of soy flour (providing 49.3 mg isoflavones) or 40 g low-fat milk powder plus 63 mg daidzein or placebo for 6 months on BP and endothelial function leading to no differences independently of the type of treatment [76].

Other RCTs focused on the investigation of the isoflavone-containing capsules on vascular outcomes, reporting contradictory findings. A double-blind, RCT evaluated the effects of genistein tablets administration (providing 54 mg/day of genistein) or placebo on 60 healthy postmenopausal women, with a mean age of 56 years, for 6 months. At the end of the treatment genistein group showed a significant increase in the brachial artery diameter (from 3.9 ± 0.8 to 4.4 ± 0.7 mm; *p* < 0.05 vs. placebo and *p* < 0.01 vs. before genistein) accompanied by an increase in the brachial arterial blood flow (from 25 ± 5 to 75 ± 7 mm; *p* < 0.05 vs. placebo and *p* < 0.01 vs. before genistein) during reactive hyperemia. No effects were observed concerning other parameters [77]. In another double-blind, RCT involving 85 postmenopausal women supplemented with 70 mg/day of isoflavone capsule (providing 38 mg glycitin, 20 mg daidzin, and 12.4 mg genistin) or placebo for 12 weeks demonstrated that isoflavone supplementation was able to decrease SBP (from 116.1 ± 14.3 to 110.8 ± 11 mmHg, *p* < 0.05) and DBP (from 74.6 ± 10 to 71.6 ± 7.7 mmHg, *p* < 0.05) in the treated group compared to baseline [78]. Other works, conducted on unhealthy participants, showed similar results. In a double-blind RCT conducted on 102 participants with prior ischaemic stroke (mean age 66 years) and supplemented for 12 weeks with 80 mg/day of isoflavone capsules (providing 80 mg purified isoflavones) or placebo, demonstrated that isoflavone consumption was correlated with a significant improvement of brachial FMD (OR 0.32, 95% CI: 0.13 to 0.80, *p* = 0.014) reversing their endothelial dysfunction status [79]. Another double-blind RCT investigated the effects of genistein tablets administration (providing 54 mg of genistein) for 6 months on 20 postmenopausal women (mean age of 58 years) with metabolic syndrome. At the end of the study, genistein-supplemented participants showed a significant increase of FMD (from 3.2 ± 4.9 to 8.9 ± 3.1) compared to the baseline (*p* < 0.001) and placebo group (*p* = 0.04) after 6 months [80]. A double-blind, RCT, including 108 postmenopausal women with metabolic syndrome, investigated the effects of 54 mg/day of genistein for 12 months: results showed a significant reduction of SBP (from 135.7 to 123.7 mmHg, *p* < 0.001 vs. baseline and *p* < 0.0002 vs. placebo group) and DBP (from 78.7 to 74.5 mmHg, *p* = 0.047 vs. baseline and *p* = 0.0541 vs. placebo group) [81]. However, other works showed opposite results. A double-blind, parallel, RCT conducted on 24 postmenopausal women with a mean age of 50 years demonstrated that 81.02 mg/day of isoflavone tablets supplementation (providing 44.02 mg daidzein, 27.08 mg glycitein and 9.92 mg genistein) for 6 weeks had no effects on BP and other cardiovascular parameters compared to the placebo group [82]. Similarly, a 2-year prospective, double-blind, RCT conducted on 431 postmenopausal women with a mean age of 57 years, and supplemented with isoflavone tablets (providing 300 mg of isoflavones), showed a slight reduction in SBP and DBP in both groups not associated with the treatment [83]. In another double-blind, RCT conducted on 50 obese postmenopausal women (mean age 60 years) supplemented with 4 isoflavone capsules daily (providing 70 mg/day of isoflavone, 44 mg daidzein, 16 mg glycitein, 10 mg genistein) or placebo for 6 months: results did not show any significant change in the treated group [84]. A double-blind, case–control study aimed to investigate the effects on cardiovascular functions of one-year genistein intervention in 22 postmenopausal women with metabolic syndrome (mean age 55 years). After the daily consumption of two tablets containing 54 mg of genistein, no significant results were detected in SBP and DBP compared to the placebo [85]. Similar results have been observed in a double-blind, RCT including 82 patients with non-alcoholic fatty liver disease (mean age of 43 years) that received 250 mg/day of genistein capsules or placebo for 8 weeks. After intervention, no differences were observed in SBP and DBP [86]. Also in another double-blind, RCT which included 38 peritoneal dialysis patients who randomly received 100 mg of soy isoflavone tablets (providing 63.72 mg genistin, 2.98 mg genistein, 26.42 mg daidzin, 3.5 mg daidzein, 2.28 mg glycitin, 1.1 mg glycitein) or a placebo for 8 weeks did not show any significant change related to treatment [87].

**Table 1:** The main characteristic of the selected randomized clinical trials concerning isoflavones and cardiovascular risk factors.

Author, Year, Country	Study Design	Participants (Mean Age)	Duration	Treatment	Isoflavones Constituent (Daily Intake)	Comparison	Main Findings
Teede, 2001, Australia [69]	Double-blind, placebo-controlled	179 healthy participants (96 men and 83 postmenopausal women) (62 y)	3 mo	56 g/d powdered soy protein isolate	118 mg isoflavones, 75.6 mg genistein, 36.96 mg daidzein, 5.04 mg glycitein	Placebo (casein)	Significant reduction in BP (*p* < 0.01) and PWV(FD) improvement (*p* = 0.02). Brachial artery FMD was significantly reduced only in men (*p* < 0.05).
Squadrito, 2002, Italy [77]	Double-blind, placebo-controlled	60 healthy postmenopausal women (56 y)	6 mo	Genistein tablets	54 mg genistein	Placebo tablets	Increase in brachial artery diameter and brachial artery blood flow (*p* < 0.01).
Sagara, 2004, UK [71]	Double-blind, placebo-controlled	50 men with relatively higher BP and/or total cholesterol (52 y)	5 wk	Diet containing at least 20 g/d of soy powder	At least 80 mg of isoflavones	Placebo diet	Decrease in SBP (*p* = 0.001) and DBP (*p* = 0.002) compared to baseline.
He, 2005, China [72]	Double-blind, controlled	302 participants untreated BP (49 y)	12 wk	40 g/d of isolated soybean protein supplement	79.4 mg total isoflavone, 44.9 mg genistein, 26.5 mg daidzein, 4.9 mg/d of glycitein	40 g/d of complex carbohydrate	Reduction in SBP (*p* = 0.01) and DBP (*p* = 0.007).
Hermansen, 2005, Denmark [73]	Double-blind, placebo-controlled	89 hypercholesterolaemic subjects (60 y)	24 wk	Soy supplement with 30 g/d soy protein 9 g/d and cotyledon fibre	100 mg isoflavones	Placebo (30 g/d casein)	No differences in FMD.
Aubertin-Leheudre, 2008, Canada [84]	Double-blind, placebo-controlled	50 obese postmenopausal women (60 y)	6 mo	Isoflavone capsules	70 mg total isoflavones, 44 mg daidzein, 16 mg glycitein, 10 mg genistein	Placebo capsules	No differences were observed.
Chan, 2008, China [79]	Double-blind, placebo-controlled	102 participants with prior ischaemic stroke (66 y)	12 wk	Isoflavone capsules	80 mg purified isoflavones	Placebo (powdered cellulose)	Improvement of brachial FMD (*p* = 0.014).
Wong, 2012, USA [82]	Double-blind, placebo-controlled	24 postmenopausal women (50 y)	6 wk	Isoflavone tables	81.02 mg total isoflavone (44.02 mg daidzein, 27.08 mg glycitein and 9.92 mg genistein)	Placebo tablets (<1.0 mg aglycone)	No effects were observed comparing treated and placebo groups.
Irace, 2013, Italy [80]	Double-blind, placebo-controlled	20 postmenopausal women with metabolic syndrome (58 y)	6 mo	Genistein tablets	54 mg genistein	Placebo tablets	Significant increase of FMD compared with placebo group (*p* < 0.001).
Kim, 2013, South Korea [78]	Double-blind, placebo-controlled	85 postmenopausal women (53 y)	12 wk	Isoflavone capsules	70 mg total isoflavone (38 mg glycitin, 20 mg daidzein, and 12.4 mg genistein)	Placebo capsules	Significant reduction of SBP and DBP compared to baseline (*p* < 0.05).
Liu, 2013, China [74]	Double-blind, placebo-controlled	180 postmenopausal women with pre or early diabetes (59 y)	6 mo	(i) 15 g soy + 100 mg isoflavones; (ii) 15 g milk protein + 100 mg isoflavone	100 mg total isoflavones (35 mg daidzein, 59 mg genistein, 4 mg glycitin	Placebo (15 g milk protein)	Subgroup analysis among pre and hypertensive women showed a significant reduction in SBP (*p* < 0.05) and sICAM1 compared to placebo group (*p* = 0.02).
Squadrito, 2013, Italy [81]	Double-blind, placebo-controlled	108 postmenopausal women with MetS (58 y)	12 mo	Genistein tablets	54 mg genistein	Placebo tablets	Significant reduction of SBP (*p* < 0.0002) and DBP (*p* = 0.0541).
Cheng, 2015, China [83]	Double-blind, placebo-controlled	431 postmenopausal women (57 y)	2 y	Isoflavone tablets	300 mg isoflavone aglycone	Placebo tablets	No differences were observed between treatment groups.
Husain, 2015, Iran [75]	Double-blind, placebo-controlled	61 postmenopausal women (50 y)	8 wk	33 g of soy in the form of biscuits	54 mg isoflavones	Placebo biscuits	No differences were observed after treatment.
Liu, 2015, China [76]	Double-blind, placebo-controlled	253 postmenopausal women (56 y)	6 mo	(i) 40 g soy flour; (ii) 40 g low-fat milk powder + 63 mg daidzein	(i) 49.3 mg isoflavones, (ii) 63 mg daidzein	Placebo (40 g low-fat milk powder)	No differences were observed after treatment.
De Gregorio, 2017, Italy [85]	Double-blind, placebo-controlled	22 postmenopausal women with MetS (55 y)	12 mo	Genistein tablets	54 mg genistein	Placebo tablets	No significant findings were found in SBP and DBP in the genistein group.
Amanat, 2018, Iran [86]	Double-blind, placebo-controlled	82 patients with NAFLD (43 y)	8 wk	Genistein capsule	250 mg genistein	Placebo capsule (cornstarch)	No differences in SBP and DBP were observed.
Movahedian, 2021, Iran [87]	Double-blind, placebo-controlled	38 peritoneal dialysis patients (soy group: 54 y; placebo group: 51 y)	8 wk	Soy isoflavone tablets	63.72 mg genistein, 2.98 mg genistein, 26.42 mg daidzein, 3.5 mg daidzein, 2.28 mg glycitin, 1.1 mg glycitein	Placebo tablets (starch)	SBP and DBP did not significantly change at the end of the treatment.
Tischmann, 2022, The Netherlands [70]	Single-blind, controlled, crossover	23 healthy volunteers (64 y)	2 × 8 wk (8 wk washout)	64 g/d soy nuts	174 mg isoflavones	No treatment	A significant increase in FMD (*p* = 0.040) was detected following the soy nut intervention compared to the placebo.

Abbreviations: BP (blood pressure); d (day); DBP (diastolic blood pressure); FD (femoro-dorsal); FMD (flow-mediated dilation); mo (month); PWV (pulse wave velocity); RCT (randomized clinical trial); SBP (systolic blood pressure); sICAM1 (soluble intercellular cell adhesion molecule 1); wk (week); y (year).

## 5. Mediating Effect of Equol-Production Status on Clinically Relevant Vascular Outcomes

The evidence from RCTs investigating the effects of soy and isoflavone supplementation on vascular outcomes indicates contrasting results. The discrepancies in the findings may be attributed, at least partially, to several factors influencing the real exposure to isoflavone metabolites. Among them, interindividual variations in the physiological response to isoflavone intake, related to the differences in the microbiota composition, and the possible interactions, including accumulating, synergistic and antagonistic effects, with other compounds from diet seems to play a major role [88].

A limited number of intervention studies explored how equolproduction status may influence the above-mentioned relation, generally reporting more pronounced effects among equol-producers compared to equol-non-producers. A double-blind RCT conducted among 190 postmenopausal women stratified into equol-producers and equol-non-producers tested the effects of 100 mg/day soy isoflavone supplementation on vascular markers. After 6 months of intervention the malondialdehyde (MDA) concentrations, an oxidative stress marker, were significantly lower in the soy-isoflavone equol-producers compared with equol-non-producers (*p* = 0.021). Although not statistically significant, similar results were also found for VCAM-1 and NO concentrations (*p* = 0.413 and *p* = 0.724, respectively) [89]. In line, another double-blind RCT comprising 202 postmenopausal women showed that 12-month supplementation with soy protein containing 99 mg isoflavones/d decreased systolic and diastolic blood pressure and improved endothelial function in the equol-producers, while when considering equol-non-producers systolic and diastolic blood pressure increased and endothelial function deteriorated during the trial [90]. A crossover RCT conducted on 60 postmenopausal women stratified based on the metabolic syndrome status and following a soy nut enriched diet for 8 weeks or control diet, reported significant reductions in diastolic BP (*p* = 0.02), TG (*p* = 0.02), C-reactive protein (CRP) (*p* = 0.01) and sICAM (*p* = 0.03) among women with MetS following soy-enriched diet, However, changes were observed only among equol-producers compared to control diet, but not among equol-non-producers. Likewise, when considering women without metabolic syndrome, only equol-producers had significant reductions in diastolic BP (*p* = 0.02) and CRP (*p* = 0.04) [91]. Among 270 equol-producing postmenopausal women a 6-month supplementation with whole soy, but not purified daidzein, decreased serum LDL-C and hs-CRP levels, when compared to the control group [92].

On the contrary, some studies exploring the effect of isoflavone supplementation on vascular inflammation markers reported no significant differences in the clinical outcomes between equol-producing and equol-non-producing individuals. In particular, a double-blind, crossover RCT comprising 117 postmenopausal women consuming either isoflavone-enriched or control cereal bars for 8 weeks did not observe any significant differences in the vascular and inflammatory markers in response to isoflavones or placebo between equol-producers and non-equol-producers [93]. Similarly, a crossover RCT conducted among 117 healthy postmenopausal women and exploring the effect of 50 mg/d isoflavone supplementation on different markers of CVD, reported no differences in response to isoflavones according to equol-production status [94]. Finally, an acute RCT conducted among male equol and non-equol-producers showed that after soy intake carotid-femoral PWV significantly improved in equol-producers at 24 h, which was significantly associated with plasma equol concentrations, while no vascular effects were observed in non-equol-producers at any time point [95].

Although the findings from the RCT suggest the differences in response to soy or isoflavone supplementation between equol-producers and nonproducers, with more pronounced clinical effects among the former, the majority has been conducted among postmenopausal women. Therefore, further research exploring the potential effects of clinically significant isoflavone doses among different populations, considering both males and females, is warranted to better elucidate the mediating effect of equol production status as well as food and dietary matrix, in the relation between isoflavone intake and vascular health.

## 6. Potential Mechanisms Mediating the Effect of Isoflavones and Gut-Derived Metabolite Equol on Endothelium

**Figure 2:**
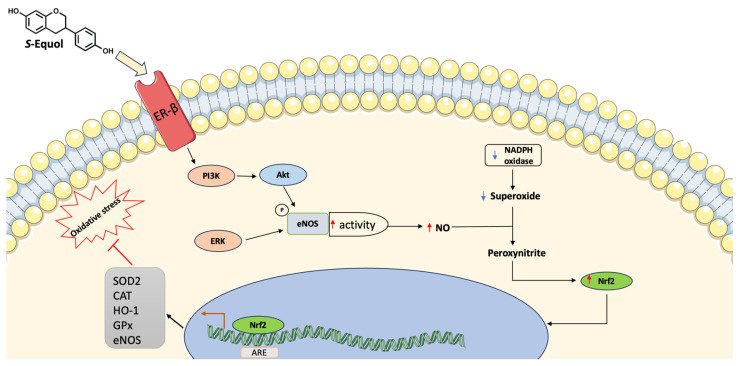
Main equol mechanisms of action in equol-producers. Equol can increase eNOS activity resulting in higher levels of NO. This can react with superoxide producing peroxynitrite and increasing Nrf2 levels leading to upregulation of antioxidant genes. ARE (antioxidant response elements), CAT (Catalase), eNOS (Endothelial nitric oxide synthase), EC (Endothelial cell), ERK (Extracellular signal-regulated kinases), GPx (Glutathione peroxidase), HO-1 (Heme oxygenase 1), NO (Nitric oxide), PI3K (Phosphatidylinositol 3-kinase), NADPH (Nicotinamide adenine dinucleotide phosphate), SOD (Superoxide dismutase). ↑ denotes increase, ↓ denotes decrease.

Isoflavones are also known as phytoestrogen for their similarity with estradiol and the ability to bind estrogen receptors (ERs) distinguishable into ER-alpha and ER-beta and distributed across different systems including the cardiovascular system [96]. Equol, the microbial-derived isoflavones metabolite, has aroused great interest for its greater positive effects on health compared to isoflavones per se (Figure 2).

Indeed, it was observed that equol has higher antioxidant activity compared to isoflavones [97,98,99] and higher bioavailability because of its reduced propensity to bind serum proteins [100]. Furthermore, equol shows an increased affinity for ER-beta compared to daidzein [101] and it is more lipophilic than isoflavones, resulting in a greater bioavailability [102]. Different studies have investigated the beneficial effects of equol on health, even if results from human studies are more inconsistent because of the great variability that distinguishes individuals in equol-producers and equol-non producers [103,104,105]. Oxidative stress and inflammation are the pivotal causes of an increased risk of developing CVD [26,27]. Increased accumulation of oxidized lipids leads to atherosclerosis through the activation of proinflammatory pathways and the increase of proinflammatory cytokines such as interleukin- (IL) 1beta, tumor necrosis factor-alpha (TNF-alpha), and nuclear factor-kB (NF-kB) [26]. Proinflammatory cytokines induce the expression of vascular cell adhesion molecule-1 (VCAM-1) by endothelial cells (ECs) increasing the adhesion of leukocytes, monocytes, and T lymphocytes to the arterial wall [106,107]. Once adhered to the endothelium, monocytes enter the vessel wall and initiate the production of monocyte chemoattractant protein-1 (MCP-1) [106] and become the mature form macrophages and begin to accumulate cholesterol into the cytoplasm transforming into foam cells [108]. Several in vivo and in vitro studies reported the anti-inflammatory effects of equol which showed the ability to reduce the expression of inflammatory biomarkers, such as prostaglandin E2 [109] and MCP-1 [110] as well as the reduction of IL-6 release and its receptor [111]. Myeloperoxidase (MPO) and nicotinamide adenine dinucleotide phosphate (NADPH) oxidase are enzymatic sources of reactive oxygen species (ROS) that lead to the production of oxidized LDL (oxLDL) [112,113]. Equol protects from oxidative stress by reducing the lipid peroxidation product malondialdehyde (MDA), enhancing the antioxidant glutathione, or increasing the activities of the enzyme superoxide dismutase (SOD) [114,115] and increasing the expression of nuclear factor erythroid 2 (Nrf2) [116]. Equol also showed the ability to reduce the NADPH-induced superoxide production [97] and to increase the expression of phosphorylated-p38 mitogen-activated protein kinase and Bcl-2 [117]. Oxidative stress and inflammation cause damage to the endothelium and alter its functions. It was observed that equol can stimulate the phosphorylation of phosphatidylinositol 3-kinase/protein kinase B (PI3K/Akt) and enhance the activity of endothelial NO synthase (eNOS) through the binding to the ER-beta receptor [118,119]. Equol can activate eNOS also via ER-independent pathways through the activation of extracellular signal-regulated kinase (ERK) 1/2 and Akt [119]. Furthermore, equol can directly upregulate eNOS transcription [120,121] while eNOS gene contains an estrogen-response element, it is reasonable that the binding of equol to ER-beta can enhance eNOS expression [122]. The NO can react with the superoxide anion producing peroxynitrite and enhancing the nuclear accumulation of Nrf2, which in turn binds the antioxidant response elements (ARE) and increases the expression of antioxidant enzymes such as superoxide dismutase, catalase, glutathione-S-transferase, glutathione peroxidase, and heme oxygenase-1 [123].

## 7. Conclusions

In vivo and in vitro studies demonstrated the beneficial effects of isoflavones and, in particular, of the microbial-derived metabolite equol [96]. The interaction with ERs seems to be the main mechanism of action that recapitulates the cardioprotective effects exerted by equol [96]. However, according to up-to-date knowledge, analysis of gut microbiota revealed that isoflavone metabolism is restricted to bacteria belonging to the *Coriobacteriaceae* family and some species that seem to have received the genes involved in these metabolic pathways through horizontal transmission [19]. Furthermore, data reported in the literature did not result in univocal findings showing that interventions with dietary food sources of isoflavone seem to have more significant results when in the context of healthier diets; similarly, supplementation of isoflavones through tablets would provide more consistent benefits among healthier individuals. These discordant results are probably due to the effect of several factors that influence the real exposure to isoflavone metabolites. Among which are the interindividual variations in the physiological response to isoflavone intake, linked with the differences in the microbiota composition, providing more beneficial effects on equol-producers compared to nonproducers. Also, the possible interactions, including accumulating, synergistic and antagonistic effects, with other compounds from diet cannot be ruled out [88]. In this regard, it is necessary to further explore the relationship between microbiota, isoflavones, and equol production to understand which other factors are involved in these differences and how it could be possible to intervene in order to spread equol beneficial effects even to equol-non producers. In this context, a more complex approach of dietary interventions with synbiotics that promote the bioconversion of isoflavones to equol is warranted.

## Data Availability

Data sharing is not applicable to this article as no new data were created or analyzed in this study.

## Abbreviations

ARE (antioxidant response elements); ARs (adrenergic receptors); BP (blood pressure); CAT (catalase); CVD (cardiovascular disease); DBP (diastolic blood pressure); DHDR (dihydrodaidzein reductase); DZNR (daidzein reductase); FMD (flow-mediated dilation); ECs (endothelial cells); eNOS (endothelial nitric oxide synthase); ERK (extracellular signal-regulated kinase); ERs (estrogen receptors); GC (guanine-cytosine); GPx (glutathione peroxidase); HO-1 (heme oxygenase 1); ICAM (intercellular adhesion molecule 1); IL (interleukin); LPS (lipopolysaccharides); MCP-1 (monocyte chemoattractant protein-1); MDA (malondialdehyde); MPO (myeloperoxidase); NADPH (nicotinamide adenine dinucleotide phosphate); NF-kB (nuclear factor-kB); Nrf2 (nuclear factor erythroid 2); O-DMA (O-desmethylangolensin); oxLDL (oxidized low-density lipoprotein); PAG (phenylacetylglutamine); PI3K (phosphatidylinositol 3-kinase); PI3K/Akt (phosphatidylinositol 3-kinase/protein kinase B); PWV (pulse wave velocity); RCT (randomized clinical trial); ROS (reactive oxygen species); SBP (systolic blood pressure); SCFAs (short chain fatty acids); SOD (superoxide dismutase); THDR (tetrahydrodaidzein reductase); TLR (Toll-like receptor); TMA (trimethylamine); TMAO (trimethylamine N-Oxide); TNF-alpha (tumor necrosis factor-alpha); VCAM-1 (vascular cell adhesion molecule-1).
